# Supplementary material for: Integrating phylogeographic and ecological niche approaches to delimitating cryptic lineages in the blue–green damselfish (Chromis viridis)
Source: PeerJ. 2019 Jul 30;7:e7384. doi: 10.7717/peerj.7384 (PMC6677123; doi:10.7717/peerj.7384)
Supplement: Supplemental Information 2 [file peerj-07-7384-s002.docx]

Table S1 The status of gene introgression in *Cytb* and Rag2 tree.

| Samples clustered in *Chromis visridis* B in *Cytb* tree | In Rag2 tree | Samples clustered in *Chromis visridis* B in Rag2 tree | In *Cytb* tree |
| --- | --- | --- | --- |
| Komo09 | *Chromis viridis* A | Komo04 | *Chromis viridis* A |
| Lz04 | *Chromis viridis* A | Lz03 | Fail to amplify |
| Lz15 | *Chromis viridis* B | Lz15 | *Chromis viridis* B |
| Lz20 | Fail to amplify | Lz40 | Fail to amplify |
| Lz47 | *Chromis viridis* B | Lz47 | *Chromis viridis* B |
| Lz50 | *Chromis viridis* B | Lz50 | *Chromis viridis* B |
| Lz51 | *Chromis viridis* B | Lz51 | *Chromis viridis* B |
| Lz52 | *Chromis viridis* B | Lz52 | *Chromis viridis* B |
| Lz54 | *Chromis viridis* A | Lz57 | *Chromis viridis* B |
| Lz57 | *Chromis viridis* B | Lz58 | *Chromis viridis* B |
| Lz58 | *Chromis viridis* B | Fiji01 | *Chromis viridis* B |
| Fiji01 | *Chromis viridis* B | Fiji03 | *Chromis viridis* B |
| Fiji03 | *Chromis viridis* B |  |  |
|  |  |  |  |
